# Conservation genomic analysis of domestic and wild pig populations from the Iberian Peninsula

**DOI:** 10.1186/1471-2156-14-106

**Published:** 2013-10-30

**Authors:** Juan Manuel Herrero-Medrano, Hendrik-Jan Megens, Martien AM Groenen, Guillermo Ramis, Mirte Bosse, Miguel Pérez-Enciso, Richard PMA Crooijmans

**Affiliations:** 1 Departamento de Producción Animal, Fac. de Veterinaria, Área de Genética, C.P.30100 Murcia, Spain; 2 Animal Breeding and Genomics Centre, Wageningen University, De Elst 1, 6708 WD Wageningen, The Netherlands; 3 Centre for Research in Agricultural Genomics, Consortium CSIC-IRTA-UAB-UB, Edifici CRAG, Campus Universitat Autonoma Barcelona, 08193 Bellaterra, Spain; 4 Institut Català de Recerca i Estudis Avançats (ICREA), 08010 Barcelona, Spain

**Keywords:** Local breeds, Population genetics, SNP, Genetic diversity, Effective population size, Pig, Iberian Peninsula

## Abstract

**Background:**

Inbreeding is among the major concerns in management of local livestock populations. The effective population size of these populations tends to be small, which enhances the risk of fitness reduction and extinction. High-density SNP data make it possible to undertake novel approaches in conservation genetics of endangered breeds and wild populations.

A total of 97 representative samples of domestic and wild pig populations from the Iberian Peninsula, subjected to different levels of threat with extinction, were genotyped with a 60 K SNP panel. Data analyses based on: (i) allele frequency differences; (ii) linkage disequilibrium and (iii) runs of homozygosity were integrated to study population relationships, inbreeding and demographic history.

**Results:**

The domestic pigs analyzed belonged to local Spanish and Portuguese breeds: Iberian ─ including the variants Retinto Iberian, Negro Iberian and Manchado de Jabugo ─, Bisaro and Chato Murciano. The population structure and persistence of phase analysis suggested high genetic relations between Iberian variants, with recent crossbreeding of Manchado de Jabugo with other pig populations. Chato Murciano showed a high frequency of long runs of homozygosity indicating recent inbreeding and reflecting the recent bottleneck reported by historical records. The Chato Murciano and the Manchado de Jabugo breeds presented the lowest effective population sizes in accordance with their status of highly inbred breeds. The Iberian wild boar presented a high frequency of short runs of homozygosity indicating past small population size but no signs of recent inbreeding. The Iberian breed showed higher genetic similarities with Iberian wild boar than the other domestic breeds.

**Conclusions:**

High-density SNP data provided a consistent overview of population structure, demographic history and inbreeding of minority breeds and wild pig populations from the Iberian Peninsula. Despite the very different background of the populations used, we found a good agreement between the different analyses. Our results are also in agreement with historical reports and provide insight in the events that shaped the current genetic variation of pig populations from the Iberian Peninsula. The results exposed will aid to design and implement strategies for the future management of endangered minority pig breeds and wild populations.

## Background

Progressive population decline has called the attention of the conservation management and scientific communities. Both for wild and domesticated populations alike there is a fear that inbreeding may lead to loss of allelic variation and adverse phenotypic consequences [1]. In addition, loss of variation may lead to reduced response to changing environments, of which genetic susceptibility to novel infectious diseases is a specific concern. Agricultural diversity in particular is of concern for future food safety [2]. Variation conserved in local breeds is often related to important traits that classically are attributed to traditional populations, such as adaptation to the environment and greater resistance to local pathogens.

In addition to these concerns, local populations are often considered to be part of the local culture and history. For instance, local pigs are often linked to local cuisine and the local landscape. The Spanish Iberico or Iberian, and Portuguese Alentejano pigs, for instance, are used to produce highly priced products due to their quality, that in part results from feeding with acorns from sparse Mediterranean oak forests, the so called 'Dehesas’. The wild relative of the pig, the wild boar, on the other hand, plays a significant role in the wildlife of the Iberian Peninsula. It is among the main prey species of an iconic predator from the Iberian Peninsula such as Iberian wolf [3]. Moreover, wild boar is an important reservoir of infectious diseases as relevant as tuberculosis in the Iberian Peninsula [4], and therefore also of concern for public and animal health.

Local pig populations, both wild and domestic, have been highly affected by human-induced changes. Local, usually fat, breeds for instance, were affected by changes in consumer preference in the middle of the 20th century when consumers started to avoid high-fat meat. As a result, a relatively small number of highly productive pig breeds progressively replaced and marginalized the traditional breeds. Many breeds became extinct in the past decades, while many other traditional breeds today face near-extinction either through dwindling population numbers or hybridization with highly productive breeds [5]. At the same time, the increase in woodland across Europe has allowed wild boar populations to increase in many countries, after having been marginalized for centuries [6]. The Iberian Peninsula provides a good representation of local pig populations, both wild and domestic. While sharing the same geography, these populations have undergone different historical events, have different phenotypic attributes, and have a different conservation status. The Iberian pigs have been reared in an extensive traditionally system in the South and West of the Iberian Peninsula for centuries, remaining isolated from the modern breeding practices developed in the late 18th and 19th century in NW Europe [7]. Iberian pigs are related to other Mediterranean pigs of Italy and The Balkans [5], which are thought to have a smaller influence from Asian pigs than the NW European pigs. Conversely, Chato Murciano and Manchado de Jabugo, now both highly endangered populations, and Bisaro resulted from crosses between native pigs from the Iberian Peninsula and foreign pigs at the end of the 19th century [8]. Beside these domestic populations, the Iberian Peninsula is also inhabited by wild boars that may represent the ancestor of these local breeds, and also constitute an important wildlife species of the Iberian Peninsula.

The recent availability of a high-density porcine SNP panel [9] provides an essential tool for genome wide association studies and genomic selection for economically important traits [10,11]. Besides the use of high-density SNP arrays for economic purposes, these panels have demonstrated their power to assess major questions in conservation genetics [12,13]. The study of linkage disequilibrium (LD) and genetic distances enable the estimation of effective population size from genetic data [14], which is of major interest in conservation genetics, especially when pedigree information is unavailable as is frequently the case for minority breeds and wild populations. In addition, high-density SNP arrays allow assessing similarities in the patterns of LD across populations (i.e. persistence of phase), providing information about the relatedness of populations [15]. The occurrence of runs of homozygosity (ROH) is indicative of demographic history and recent inbreeding [13,16]. While the same parameters can be interpreted as signatures of selection on genomic regions [17,18], when taken as global genomic parameters, they are highly indicative of demographic history [19], if properly corrected for local recombination rate [13]. A genome-wide SNP assay, combined with a detailed recombination map for the species [20] can therefore aid in giving insight into the conservation management of pig populations. Despite the fact that SNP assays are gaining interest for traceability purposes [21,22], only few studies have used a high-density SNP assays for conservation purposes [1,12,23-25].

Here we present a comprehensive study in which high-density SNP data from domestic and wild pigs were used to address questions important to conservation genetics. First, we assessed the relationships between pigs by population structure analysis and by investigating the persistence of LD phase. Secondly, patterns of LD in each population were used together with a high-density recombination map to estimate past and present effective population size. Finally, the number and size of ROH were investigated in each individual. The joint analysis of all those parameters allowed us to obtain reliable and consistent data of population structure, inbreeding and demographic history in each population providing valuable insights for future management strategies in pig from the Iberian Peninsula.

## Results

A total of 97 pigs from domestic and wild autochthonous populations from the Iberian Peninsula were genotyped with the Porcine SNP60 Beadchip [9]. The SNPs located on the sex chromosomes and those with more than 5% missing genotypes were excluded from the analysis, resulting in a total of 47,594 SNPs used for the analysis.

### Population structure

**Figure 1:**
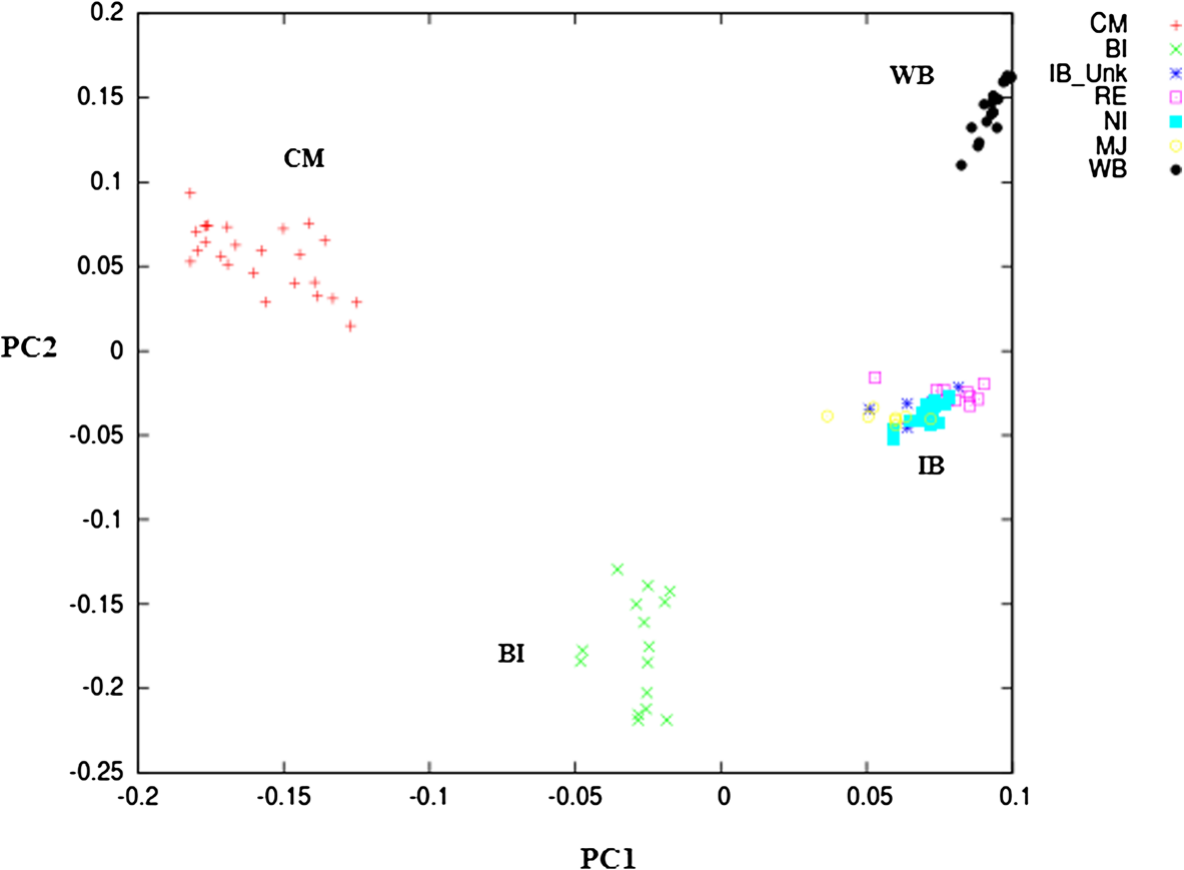
**Different population groups defined with PCA analysis.** WB, wild boar; IB, Iberian pig; IB_Unk, Iberian unidentified variant; NI, Negro Iberian; RE, Retinto; MJ, Manchado de Jabugo; BI, Bisaro; CM, Chato Murciano.

The Principal Component Analysis (PCA) revealed four main clusters represented by wild boar, Iberian, Bisaro and Chato Murciano (Figure 1). Among populations, Chato Murciano was the most divergent breed, showing a pairwise F_st_ ≥ 0.22 with all populations except for Bisaro where it was 0.18. The variants of Iberian ─Retinto, Negro Iberian, Manchado de Jabugo and five unclassified Iberian pigs─ showed low Nei’s genetic distances between them (≤ 0.06) and low F_st_ ( ≤ 0.05), and likewise the two populations of wild boar from Spain and Portugal (0.04 and 0.06 respectively). Among domestic pigs, Iberian variants showed the lowest Nei’s genetic distance to wild boar (0.10 - 0.12). The results from Nei’s genetic distances and the pairwise Fst between populations are detailed in Additional file 1.

**Figure 2:**
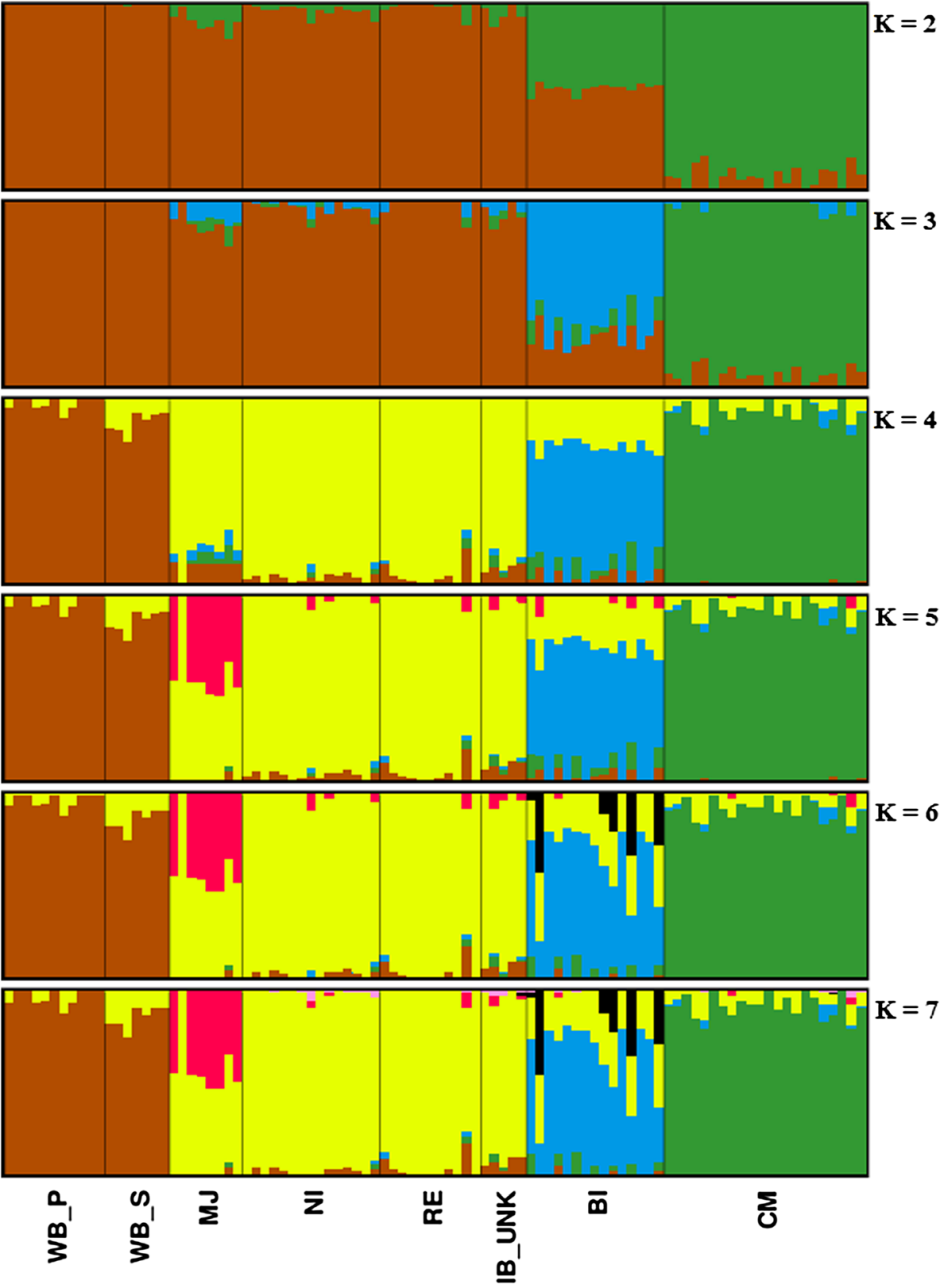
**Graphic representation of estimated membership coefficients for each individual for K =2-7.** Each color represents the proportion of the genome assigned to each assumed cluster.

The Bayesian clustering algorithms implemented in the Structure software assigned all individuals to clusters that coincide to their population of origin, with the exception of one Manchado de Jabugo pig (MJ_02) that was placed in the cluster of the other Iberian variants. Pairwise genetic distances between individuals (Additional file 2) and PCA analysis done using the Iberian pig data only (Additional file 3) confirmed this finding. K values from 2 to 7 were tested (Figure 2). The optimal K-value was estimated using the method described by Evanno et al. [26], indicating that K = 4 was the most parsimonious number of clusters (Additional file 4) in full agreement with the PCA analysis. Chato Murciano and Bisaro appeared as differentiated clusters when K = 2-3, while Iberian and wild pigs shared the same cluster for those K values. The Iberian cluster (yellow) contributed to all the other populations, in particular to Bisaro (25%) and wild pigs from Spain (12%) (Additional file 4). No differentiation between wild boar from Portugal and Spain was apparent, nor between the Iberian, Retinto and Negro Iberian variants, for any of the K values tested. However, signs of admixture from an unspecified origin were observed in Manchado de Jabugo for K ≥ 5. Thus, for subsequent analysis, samples were grouped as follows: Bisaro, Chato Murciano, Manchado de Jabugo, Iberian pig and wild boar. In addition, the Iberian variants Retinto and Negro Iberico were also analyzed separately. Finally, for population-based analyses the pig MJ_02 was removed since it fell outside of any of the groups considered in the analysis.

### Linkage disequilibrium among populations

**Figure 3:**
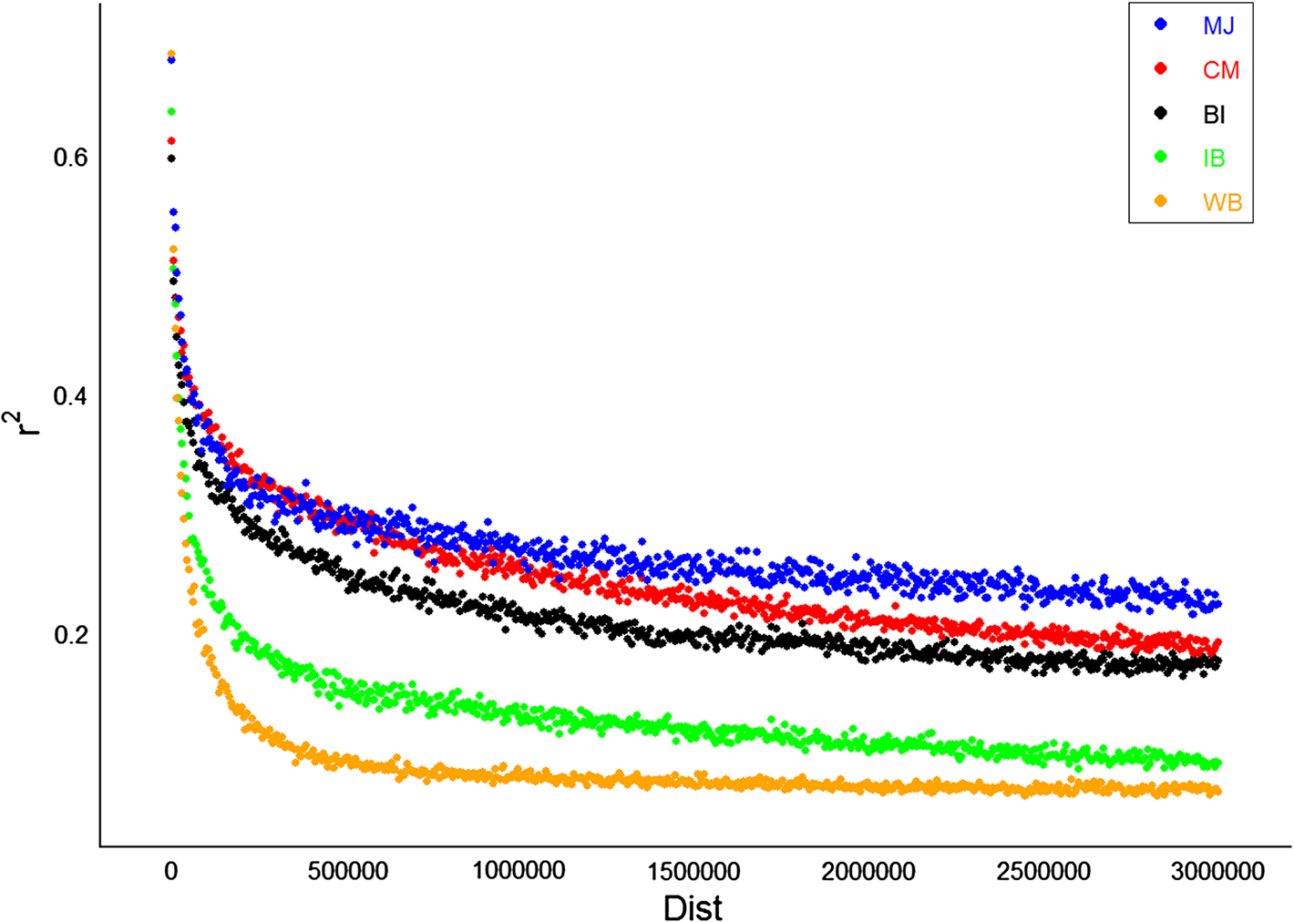
**Decline of LD measure by r2 against distance in bases across the 18 autosomes*.*** WB, wild boar; IB, Iberian; MJ, Manchado de Jabugo; BI, Bisaro; CM, Chato Murciano.

A total of 24,703 SNPs in wild boar, 29,856 SNPs in Bisaro, 33,454 in Chato Murciano, 27,858 in Iberian and 26,246 in Manchado de Jabugo were used to estimate LD for all SNP pairs less than 3 Mbp apart (Table 1). Pairwise r^2^ values were averaged over all 18 autosomes and plotted as a function of increasing genetic distance in all populations studied (Figure 3; Additional file 1). The persistence of LD as the distance between loci increased and the strength of LD, varied widely between populations and between chromosomes. The decay of LD as a function of marker distance was greater in wild boar (r^2^ < 0.2 within 0.1 Mbp) than in the domestic breeds and showed the lowest average r^2^ across all genetic distances. Among the domestic breeds, LD was the lowest in Iberian (r^2^ < 0.2 within 0.2 Mbp). By contrast, Manchado de Jabugo and Chato Murciano had the most pronounced extent of LD at short genetic distances, although LD decreased faster in Chato Murciano than in Manchado de Jabugo for genetic distances higher than 1 Mbp.

**Table 1:** **Linkage disequilibrium (r**^**2**^**) and recombination rate averaged per chromosome and per population**

**Chrom**	Rec. rate (cM/Mb)*	**r**^**2**^ **± SD**				
		CM	BI	IB	MJ	WB
1	0.36	0.29 ± 0.27	0.25 ± 0.24	0.13 ± 0.15	0.26 ± 0.29	0.11 ± 0.15
2	0.64	0.22 ± 0.24	0.24 ± 0.22	0.16 ± 0.17	0.3 ± 0.31	0.10 ± 0.13
3	0.71	0.25 ± 0.25	0.17 ± 0.19	0.12 ± 0.13	0.26 ± 0.29	0.09 ± 0.13
4	0.67	0.27 ± 0.27	0.18 ± 0.20	0.12 ± 0.12	0.24 ± 0.26	0.11 ± 0.13
5	0.86	0.25 ± 0.25	0.20 ± 0.21	0.11 ± 0.13	0.25 ± 0.28	0.09 ± 0.12
6	0.80	0.26 ± 0.23	0.22 ± 0.23	0.15 ± 0.17	0.27 ± 0.29	0.10 ± 0.14
7	0.85	0.24 ± 0.24	0.21 ± 0.22	0.12 ± 0.12	0.30 ± 0.31	0.09 ± 0.12
8	0.65	0.28 ± 0.26	0.24 ± 0.23	0.15 ± 0.17	0.24 ± 0.27	0.11 ± 0.14
9	0.73	0.24 ± 0.25	0.24 ± 0.23	0.12 ± 0.13	0.30 ± 0.30	0.09 ± 0.12
10	1.14	0.24 ± 0.25	0.18 ± 0.20	0.09 ± 0.11	0.23 ± 0.27	0.08 ± 0.10
11	0.75	0.23 ± 0.23	0.19 ± 0.20	0.16 ± 0.18	0.26 ± 0.26	0.10 ± 0.12
12	1.24	0.26 ± 0.24	0.20 ± 0.20	0.13 ± 0.13	0.24 ± 0.27	0.09 ± 0.12
13	0.46	0.30 ± 0.26	0.27 ± 0.26	0.17 ± 0.19	0.36 ± 0.29	0.11 ± 0.15
14	0.73	0.30 ± 0.26	0.24 ± 0.24	0.17 ± 0.18	0.30 ± 0.28	0.09 ± 0.12
15	0.61	0.30 ± 0.26	0.21 ± 0.22	0.14 ± 0.15	0.26 ± 0.27	0.11 ± 0.14
16	0.78	0.31 ± 0.26	0.21 ± 0.21	0.13 ± 0.15	0.27 ± 0.29	0.10 ± 0.13
17	0.95	0.27 ± 0.26	0.22 ± 0.23	0.11 ± 0.13	0.24 ± 0.29	0.09 ± 0.11
18	0.81	0.21 ± 0.22	0.20 ± 0.20	0.09 ± 0.12	0.23 ± 0.28	0.09 ± 0.12
Total	0.76	0.26 ± 0.25	0.22 ± 0.22	0.13 ± 0.14	0.27 ± 0.28	0.10 ± 0.13

*Averaged recombination rate among 4 pig populations [20].

### Persistence of phase

**Figure 4:**
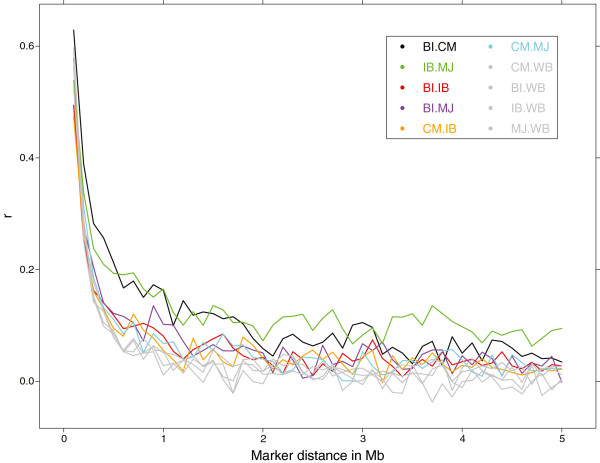
**Correlation of Phase between populations for SNP pairs grouped by distance across the whole genome.** The pairs between wild boar and domestic pigs (WB – IB/MJ/CM/BI) were uniformly plotted in gray for ease of reading. WB, wild boar; IB, Iberian; MJ, Manchado de Jabugo; BI, Bisaro; CM, Chato Murciano.

The persistence of LD phase was calculated as the Pearson correlation (r) between SNP pairs in all possible population pairs. Similar to LD, r decreased as the distance between markers increased. This was observed for all pairs of populations, although at different degrees (Figure 4; Additional file 5). Bisaro and Chato Murciano showed the greatest correlation of phase at short genetic distance. However, for SNP pairs spaced more than 1.5 Mb apart, Iberian and Manchado de Jabugo showed the highest correlation of phase. Correlations between the other pairs of domestic pig populations (CM-MJ, CM-IB, BI-IB, BI-MJ) tended to be similar. The persistence of phase found between wild boar and all domestic pigs was lower than between domestic populations.

### Current effective population size

The mean values of LD for all 1 Mb bins across the entire genome were used to estimate the current effective population size (N_e_) implementing the equation r^2^ = 1/(4N_e_c +1) [27]. This estimation was performed taking into account the recombination for each of these bins [20]. Large N_e_ was observed for the wild boar population (Table 2). Among the domestic breeds, Iberian also had a high N_e_ (N_e_ = 151 ± 84) while Manchado de Jabugo and Chato Murciano had smaller effective population sizes (N_e_ = 46 ± 50 and 59 ± 31 respectively). Nevertheless, notice the large SD of these estimates.

**Table 2:** **Current Effective population size (N**_**e**_**) in each population. Sample size (N); Standard deviation (SD)**

**POP**	**N**	**Ne ± SD**
BI	15	74 ± 37
CM	25	59 ± 31
MJ	7	46 ± 50
NI	15	95 ± 49
RE	10	88 ± 126
IB*	31	151 ± 84
WB	18	180 ± 61

*Ne in Iberian Breed, considering RE, NI and IB_Unk as a single population.

### Past effective population size

**Figure 5:**
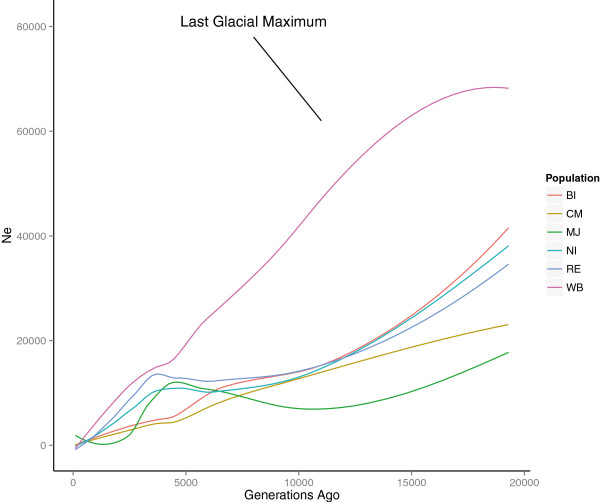
**Estimated effective population size (Ne) over time.** WB, wild boar; NI, Negro Iberian; RE, Retinto; MJ, Manchado de Jabugo; BI, Bisaro; CM, Chato Murciano.

The past N_e_ at generation T, where T = 1/2c [14], was similarly estimated for each bin of 1 Mb and sorted based on decreasing recombination rate values. This approach allowed studying N_e_ from as few as 5 to 20,000 generations ago (Figure 5). Similar to the estimation of the current N_e_, wild boar tended to have the highest past N_e_, followed by Iberian pigs. A noteworthy drop in N_e_ was observed in wild boar 10,000 – 20,000 generations ago, with a decrease of N_e_ from over 70,000 to below 30,000. The N_e_ increased rapidly in Iberian pigs at ~3,500 - 5,000 showing a maximum N_e_ at ~3,500 generations ago (N_e_ ~ 12,000). This increase in N_e_ was not observed in any other population (Additional file 6).

### Runs of homozygosity

**Figure 6:**
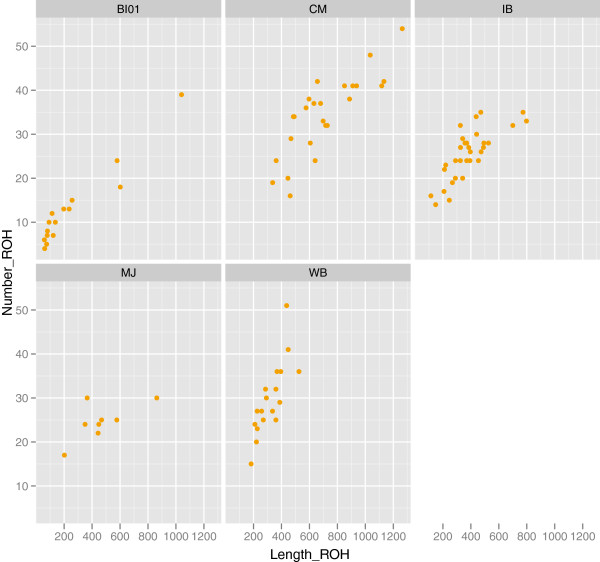
**Average of number of ROH Vs. average of length of ROH.** Each dot represents an individual. WB, wild boar; IB, Iberian; MJ, Manchado de Jabugo; BI, Bisaro; CM, Chato Murciano.

ROH of a minimum of 10 kbp containing at least 20 homozygous SNPs were studied in each individual separately. All individuals included in this study showed ROH. However, there were marked differences between populations in terms of number and length of ROH (Figure 6). The sums of all ROH per animal allowed the estimation of the percentage of the genome covered by ROH in each population (Additional file 7). The Chato Murciano had the largest mean proportion of its genome covered by ROH (29%). Other populations had mean values lower than 20%, with Bisaro displaying the lowest mean proportion (10%). The mean of the total number of ROH per population was higher in Chato Murciano and wild boar (34 and 30 respectively) than in Iberian and Manchado de Jabugo (26 and 24 respectively). The Bisaro breed showed the lowest mean number of ROH (13). Regarding the length of ROH, approximately 36% of the Chato Murciano pigs analyzed had long ROH (> 100 Mbp) and 92% of the pigs of this breed had ROH in the range of 50–100 Mbp, making Chato Murciano the population with the highest proportion of long ROH. By contrast, none of the wild boars analyzed had long ROH, and only 20% of wild pigs analyzed contained ROH in the range of 50–100 Mbp, indicating that wild boar had shorter ROH than the other populations. Manchado de Jabugo and Bisaro contained fewer ROH than the other populations, with Manchado de Jabugo displaying a higher proportion of long ROH than Bisaro. Twenty-five percent of Manchado de Jabugo pigs showed long ROH and 75% contained ROH in the range of 50–100 Mbp. These percentages are 6% and 33% respectively in Bisaro. Finally, Iberian had values intermediate to these breeds, since 16% of Iberian pigs displayed long ROH and 58% in the range of 50 – 100 Mb. All the pigs analyzed showed ROH shorter than 50 Mbp.

A Pearson’s correlations matrix was made including LD, length of ROH and recombination rate. We found a positive correlation between mean values of LD and length of ROH per chromosome (ρ = 0.70, p < 0.002) while the correlation was negative between lengths of ROH and recombination rates (ρ = - 0.67, p < 0.003).

## Discussion

High density SNP analysis can provide information on past and current population demography. LD is largely affected by population history and demography [28-30], constituting a potential tool to be applied to genetic population management. Specifically, LD can be used to estimate past and present effective population size [31] and to study persistence of LD phase [24]. The availability of a large number of SNPs, allows the study of parameters that can be directly relevant to assess effects of inbreeding such as the occurrence of Runs of Homozygosity (ROH). In addition, high-density SNP arrays are expected to improve the accuracy to assess population structure and relationship among populations [32-34]. Nevertheless, the applications of high-density SNP assays for investigations into genetic population management of minority breeds and wild populations in pig are scarce.

### Relationships between populations

Understanding the relationships among and within populations of livestock is a necessary first step to establish conservation priorities and strategies [35,36]. Population structure analysis, based on differences in allele frequencies, has been a proven method to assess relationships between populations [23,37]. We combined this widely used approach with the estimates of persistence of phase as a measure of relationship between populations [24]. The different methods implemented to assess the relationships between populations showed a high degree of congruence.

The results obtained from the population structure and persistence of phase analyses indicate closer relationships between Chato Murciano and Bisaro, and between Iberian pigs and wild boar. This observed division seems to reflect the classical separation of pig populations from the Iberian Peninsula into two origins: the Celtic type and the Iberian type pigs [5]. Most of Celtic type breeds from the Iberian Peninsula are now extinct or are highly endangered. The Bisaro pig is a representative of this group [38]. All the variants of Iberian, which include Retinto, Negro Iberico and Manchado de Jabugo, belong to the Iberian type. Although the similarity between Chato Murciano and Bisaro could be due to a Celtic origin ─ or at least a mixed origin ─ of Chato Murciano, it is possible that the differentiation between the two groups of pigs actually differentiate admixed and non-admixed populations.

The high Pearson correlations for persistence of phase at long genetic distances detected between Manchado de Jabugo and other Iberian variants is typical for subpopulations of the same breed [15]. Furthermore, structure analysis confirmed the close genetic relationship between variants of Iberian pig but also signs of genetic admixture in Manchado de Jabugo. This is agreement with historical records documenting that Manchado de Jabugo is a variant of the Iberian crossed with foreign pigs [8].

### Inbreeding and effective population size

The analysis of N_e_ and ROH can be used to address major issues in conservation genetics such as effects of genetic drift and inbreeding [39]. The small population size inferred for the majority of local populations enhances the effect of consanguinity and genetic drift, which could compromise the long-term viability of the populations [40]. With the absence of pedigree data in many local breeds, genetic marker data can be used as a surrogate to estimate current and past N_e_, for instance through exploring the extent of LD [31]. Despite the interest in N_e_ for conservation of populations, the estimation of this parameter is remarkably complex [41]. The estimation of N_e_ assumes an ideal population that is isolated, without migration, with random mating and with constant linear population growth [14]. Although it is recognized that these assumptions are generally violated in natural populations, estimation of N_e_ is widely used. The estimation of recent N_e_ has been computed using linked [31,42] and unlinked [43] genetic markers. We estimated N_e_ separately for 1 Mb bins containing information of recombination rates in order to obtain more information of demographic history [42]. While this method may provide a greater temporal dimension of N_e_[44] it may make it difficult to interpret the results of current N_e_[41]. Additionally, our estimate of current N_e_ must be treated with care due to the low sample sizes, especially in those populations with a sample size lower than 15 animals (i.e. Manchado de Jabugo and Retinto).

ROH have been used to infer the history of consanguinity in human populations [39,45] and cattle [16]. These studies have demonstrated that long ROH are related with high consanguinity levels and also have shown the existence of a good correlation with pedigree inbreeding coefficients [16,46].

The existence of Chato Murciano pigs with a high number of long ROH shows the importance of recent inbreeding and thus low individual genetic diversity. Indeed, we observed three Chato Murciano pigs that had more than 45% of the genome covered by ROH, but also pigs with much lower percentage. This observation is consistent with known management strategies of this breed [23] and in agreement with a strong bottleneck described for this breed about 20 years ago when the entire breed consisted of only 30–40 breeding animals. The high number of long ROH also indicates that this breed has not recently been extensively crossed with other breeds otherwise the long ROH would have broken down. The frequency of long ROH in Manchado de Jabugo was similar to the other Iberian variants. Recent admixture between Manchado de Jabugo and other pig breeds, as observed in the structure analysis, may have resulted in the break-down of long (>100 Mbp) homozygous haplotypes. Despite the fact that Manchado de Jabugo is highly endangered with extinction as suggested from the small census population size (http://dad.fao.org/), this population did not show signs of high levels of consanguinity, likely because of its admixed origin. Thus, the conservation program currently implemented in Manchado de Jabugo is effective and necessary to assure the future viability of this population. What is also evident, however, is that this management strategy has gone at the expense of the historical genetic distinctiveness of the breed. By contrast, Bisaro showed signs of low consanguinity in agreement with its mixed origin and the strict conservation program implemented in this breed [47]. Although the Iberian pigs generally showed relatively low percentage of the genome covered by ROH, a few individuals showed a high coverage by ROH [48]. This can be expected in this heterogeneous breed which consists of local populations and different color forms.

The agreement between our observations and expectations based on historic reports highlights that analyzing the structure of ROH can aid in assessing levels of current consanguinity, and historic events such as bottlenecks in local pig populations. Furthermore, the assessment of ROH at the individual level has practical implications in conservation programs. Animals displaying high levels of ROH, for instance, could be excluded or given lower priority for breeding purposes in endangered populations. However, it must be taken into account that the 60 K SNP panel applied may underestimate the number of small ROH due to ascertainment bias [13] and may inflate the length of the longest ROH [16]. Yet Bosse *et al*. [13] and Purfield *et al*. [16] concluded that high-density SNP panels allow an appropriate estimation of ROH, especially for the analysis of large ROH.

### Demographic history

The study of demographic history provides a better understanding of the current risk of inbreeding, and might facilitate predicting the effects of future changes in effective population size. Despite the fact that estimation of demography implies the simplification of a complex biological reality, estimation of effective population size based on LD and recombination rate provides useful predictions and consistent comparisons between populations [31,49]. It must be considered that the estimation of recent N_e_ is more inaccurate than the estimation ancient N_e_ owing to the increase in the variability of N_e_ values as the length of the segment used for the estimation increase [14]. The estimation of past N_e_ for wild boar tended to be higher than for domestic populations, with important drops in N_e_. Moreover, wild boar had a very high number of short ROH and no long ROH. A high number of short ROH has been related with a reduced population size in the past and low inbreeding in recent times [39]. This pattern could be explained by the bottlenecks that occurred in Europe in the last century [50], that would have reduced the N_e_ of wild boar drastically. Moreover, continuous events of formation of subpopulations and migration between them, favored by the lack of geographic barriers across the Iberian Peninsula, and even occasional admixture with domestic pigs, could have avoided high inbreeding in wild boar populations. Genetic signs of migration between subpopulations of Iberian wild boars have been described in Portugal [51].

The low recombination rate observed in large parts of the porcine genome essentially allows a much wider window in the past effective population size. Assuming a generation interval of approximately 2 years [24]. We observed a distinct drop of the N_e_ between 20,000 and 10,000 generations ago exclusively in wild boar that seems to reflect the sharp population decrease during the Last Glacial Maximum [52]. The increase in population size observed exclusively in Iberian pigs around 4000–4500 generations ago (see Additional file 6) is consistent with the time frame of the domestication in Europe [53,54]. Recently the role of Europe as domestication centre has been dismissed [53,54]. In this scenario of domestication, European domestic pigs appeared as a result of repeated events of admixture between domestic pigs imported from Near Eastern regions around 8,000 years ago [55] and wild pigs from Europe [53]. On one hand, the fact that Iberian pig allowed the study domestication events implies that this breed represents a suitable model to study domestication in Europe, reinforcing the need to preserve the breed and avoid admixture with other pig populations. On the other hand, it confirms historic reports and previous studies with mtDNA showing that Iberian pigs did not originate from crosses with other breeds [56]. Admixture events may mask genetic signs of past demographic events [54] explaining why other domestic breeds such as Bisaro and Chato Murciano showed a different pattern of past N_e_. Studies using Next Generation sequence data are needed to support and increase the accuracy of past N_e_ estimations in Iberian pig.

Structure analysis showed that Iberian pigs contributed to the wild boar genetic stock (Figure 2). This fact together with the genetic distances and F_st_ values between wild boar and Iberian pigs provide support that Iberian may have been crossbreeding with wild boar until medieval times [57]. This is in agreement with results for the European breeds studied by Groenen *et al.*[52], who describe a complex history in European breeds and incomplete lineage sorting, supporting admixture between wild and domestic pigs in Europe. It must be kept in mind that Iberian pigs were traditionally bred outdoors, which has enabled crossbreeding between wild and domestic pigs. Intriguingly, this is unlikely to have affected only the domesticated pigs, but rather also the wild boar of the Iberian Peninsula, as suggested by recent investigations on introgression from domesticated into wild populations [12].

## Conclusions

This study provides a comprehensive picture of demographic history, population structure and inbreeding of wild and domestic pig populations from the Iberian Peninsula as well as their relevance in conservation genetics. The occurrence of ROH in Chato Murciano was very high in some individuals, which may be due to a recent bottleneck and also highlights the lack of a well-designed genetic management program. Manchado de Jabugo showed a relatively high heterozygosity. This is unexpected given the extremely low census population size, and most likely reflects recent admixture with commercial pig breeds observed in this population. Conservation programs need to be maintained and carefully designed in order to avoid further loss of genetic distinctiveness. The study of N_e_ and ROH in Bisaro indicated high genetic diversity of this breed as a result of its mixed origin and the efforts carried out to preserve this breed. We observed that the Iberian breed may represent a good model to assess genetic signs of past demographic events as domestication, being an additional argument for the need to preserve Iberian pig breed and to avoid crossbreeding with other breeds. Previous evidence supporting the Iberian breed as being closely related to wild boar were confirmed and further evidence was provided for recurrent crossbreeding between these populations in the past. The analysis of wild populations from different regions of the Iberian Peninsula indicates natural migrations of wild pigs across the Iberian Peninsula as well as low levels of inbreeding in Iberian wild boar.

## Methods

### Animals and sampling

A total of 97 unrelated pig samples were collected from populations of the Iberian Peninsula, and genomic DNA was extracted by standard protocols. The study included 18 wild boars (WB) from different regions of Portugal (n = 11) and Spain (n = 7), and 79 domestic pigs. The domestic pigs utilized for the analysis belonged to three local breeds: Iberian ─including a Retinto Iberian variant (RE, n = 11), a Negro Iberian variant (NI, n = 15), an unidentified Iberian variant (IB, n = 5), and Manchado de Jabugo (MJ, n = 8) ─, Bisaro (BI, n = 15) and Chato Murciano (CM, n = 25).

### SNP genotyping

High-density SNP genotyping was performed using the Porcine SNP60 Beadchip (IlluminaInc, USA) designed to genotype 62,163 SNPs [9], according to manufacturer’s protocol. For this study, only SNPs mapped to one of the 18 autosomes on *Sus scrofa* build 10.2 and with less than 5% missing genotypes were included in the analysis.

### Data analysis

Allele Sharing Distances were calculated using PLINK v1.07 [45]. Nei’s genetic distances [58] and F_st_ values between populations were calculated using the Power marker software [59]. The pairwise distances between individuals were used to construct a Neighbor-Joining tree in Mega 5.03 [60]. The admixture model implemented by the program *Structure* v2.0 [61] was used to examine relatedness among pig populations and population stratification. K values (number of assumed clusters) from two to seven were tested. Consistent results were obtained by using a burning period of 100,000 followed by 100,000 Markov chain Monte Carlo (MCMC) repetitions. The analysis was replicated and the most likely number of clusters was determined by the Evanno method [26] using the web server Structure Harvester [62]. Moreover, to obtain further detail of the population structure, the PCA was performed using the program Eigenstrat [[63]].

#### Linkage Disequilibrium analysis

Markers significantly deviating from Hardy-Weinberg equilibrium (P < 0.001) and with a MAF lower than 0.05 were excluded from LD analysis using PLINK v1.07 [64]. LD (r2) was estimated for all marker pairs less than 3Mbp apart across all populations and in each autosomal chromosome independently using Haploview 4.2 [65]. Graphic display of r^2^*vs.* distance per chromosome and means plot of r^2^ in each breed vs. each chromosome were made in R environment (http://www.r-project.org/).

#### Persistence of phase

To calculate the persistence of phase and the time since two breeds diverged we followed the procedure implemented by Badke *et al.*[24]. Briefly, the SNP data was split into groups of SNPs with pairwise marker distance of 100 kbp, and the pairwise Pearson correlation between SNP was estimated across the 10 possible pairs of population.

#### Effective Population size

Effective population sizes were calculated in all populations implementing the equation r^2^ = 1/(4N_e_*c* +1) → N_e_ = (1/4c) * (1/r^2^ -1), where r^2^ is the LD, *c* is the marker distance in Morgans between SNP and N_e_ is the effective population size [27]. Additionally, past effective population size at generation T was calculated by the approximation T = 1/2*c*[14]. This formula implies that regions of low recombination rate allow the study of ancient N_e_.

Previous authors [25] tended to apply the generalization 1 Mb ~ 1 cM to calculate N_e_, but this assumption may lead to incorrect estimates of N_e_. Recombination rate varies considerably across and within porcine chromosomes [20], to an even larger extent than observed in other mammals [13]. Instead, we used the averaged high-density recombination map described by Tortereau *et al.*[20]. The effective population size estimates were derived by averaging multiple genomic regions in order to have a better approximation of the effective population size [49]. Towards this end, the chromosomes were divided in 1 Mb bins containing information of recombination rates and average r^2^ for all possible pairs of SNPs included in each bin. Mean values and standard deviations among bins were subsequently used to estimate past and present effective population size. The approximation of past N_e_ assumes that *c* is much larger than the mutation rate (~10^-8^ per locus and generation) [15] so bins with *c* < 10^-6^ where not considered for past N_e_ estimation.

#### Runs of homozygosity

The software PLINK v1.07 [64] was used to detect ROH for individuals separately. The ROH were defined by a minimum of 10 kbp in size and 20 homozygous SNPs. One heterozygous SNP was permitted in ROH, so that the length of the ROH was not disrupted by an occasional heterozygote. In addition, minimum SNP density of 1 SNP/Mb and a largest possible gap between SNPs of 1 Mb were predefined in order to assure that the ROH were not affected by the SNP density.

Number of ROH, total length of ROH and the average of ROH length in each animal were calculated for each chromosome and the mean across animals was estimated for each breed. Those ROH longer than 100 Mbp were categorized as long ROH. The percentage of the total genome length affected by ROH in each animal was also inferred.

### Availability of supporting data

The data sets supporting the results of this article are included within the article (and its additional files).

## Competing interests

The authors declare that they have no competing interests

## Authors’ contributions

JMHM analyzed the data and wrote the manuscript. HJM designed and conceived this study. MAMG critically review the manuscript. MB designed the ROH analysis and supervised the corresponding writing parting of the manuscript. MPE collected samples and helped draft the manuscript. RPMAC and GRV collected the genetic data and participated in the supervision of the study. All authors read and approved the final manuscript.

## Supplementary Material

Additional file 1: Table S1 F_st_ pairwise (below the diagonal) and Nei’s genetic distances (above the diagonal) between populations. Table S2: Average r^2^ value for SNP spaced 0.5, 1.0, 1.5, 2.0, 2.5 and 3.0. Click here for file

Additional file 2 **Neighbor-Joining tree of pig populations constructed from individual pairwise genetic distance.** WBPOR, wild boar from Portugal; WBSPN, wild boar from Spain; RE, Retinto Iberian; NI, Negro Iberian, IB, Iberian (unidentified variant); MJ, Manchado de Jabugo; BI, Bisaro; CM, Chato Murciano. Click here for file

Additional file 3 **PCA analysis using exclusively Iberian pigs.** IB_Unk, Iberian unknown variant; NI, Negro Iberian; RE, Retinto; MJ, Manchado de Jabugo. Click here for file

Additional file 4 STRUCTURE analysis and Evanno method to determine the optimal number of clusters; Membership coefficient of the breeds tested in the four clusters inferred by STRUCTURE software. Click here for file

Additional file 5 Persistence of phase for intervals of 100 kb ranging from 0 to 10 Mb. Click here for file

Additional file 6 **Estimated effective population size (N**_e_) over time: detail of 10,000 generations ago. WB, wild boar; NI, Negro Iberian; RE, Retinto; MJ, Manchado de Jabugo; BI, Bisaro; CM, Chato Murciano. Click here for file

Additional file 7 **Estimation of the percentage covered by ROH in each population.** WB, wild boar; IB, Iberian; MJ, Manchado de Jabugo; BI, Bisaro; CM, Chato Murciano. Click here for file
