# Supplementary material for: Conservation genomic analysis of domestic and wild pig populations from the Iberian Peninsula
Source: BMC Genet. 2013 Oct 30;14:106. doi: 10.1186/1471-2156-14-106 (PMC3840735; doi:10.1186/1471-2156-14-106)
Supplement: Additional file 1: Table S1 — Fst pairwise (below the diagonal) and Nei’s genetic distances (above the diagonal) between populations. Table S2: Average r2 value for SNP spaced 0.5, 1.0, 1.5, 2.0, 2.5 and 3.0. [file 1471-2156-14-106-S1.pdf]

Table S1. F_{st} pairwise values (below the diagonal) and Nei's genetic distances (above the diagonal) between populations.

	BI	CM	IB_UNK	MJ	NI	RE	WB_P	WB_S
**BI**	0,000	0,117	0,102	0,106	0,095	0,103	0,133	0,134
**CM**	0,182	0,000	0,145	0,148	0,139	0,148	0,176	0,176
**IB_UNK**	0,133	0,221	0,000	0,069	0,036	0,040	0,108	0,107
**MJ**	0,158	0,239	0,098	0,000	0,058	0,065	0,117	0,117
**NI**	0,157	0,237	0,032	0,102	0,000	0,033	0,101	0,101
**RE**	0,172	0,254	0,046	0,123	0,055	0,000	0,103	0,102
**WB_P**	0,219	0,292	0,201	0,228	0,204	0,218	0,000	0,045
**WB_S**	0,177	0,260	0,158	0,190	0,169	0,186	0,056	0,000

Table S2. Average LD (r^2) for SNP spaced 0.5, 1.0, 1.5, 2.0, 2.5, 3.0 in each population.

Genetic Distance	CM	BI	IB	MJ	WB
**0.5 Mb**	0.29	0.25	0.16	0.29	0.10
**1.0 Mb**	0.26	0.22	0.13	0.27	0.08
**1.5 Mb**	0.22	0.19	0.12	0.26	0.07
**2.0 Mb**	0.21	0.18	0.10	0.25	0.07
**2.5 Mb**	0.20	0.17	0.09	0.23	0.07
**3.0 Mb**	0.19	0.18	0.09	0.22	0.07
