# Supplementary figures and images for: Conservation genomic analysis of domestic and wild pig populations from the Iberian Peninsula
Source: BMC Genet. 2013 Oct 30;14:106. doi: 10.1186/1471-2156-14-106 (PMC3840735; doi:10.1186/1471-2156-14-106)

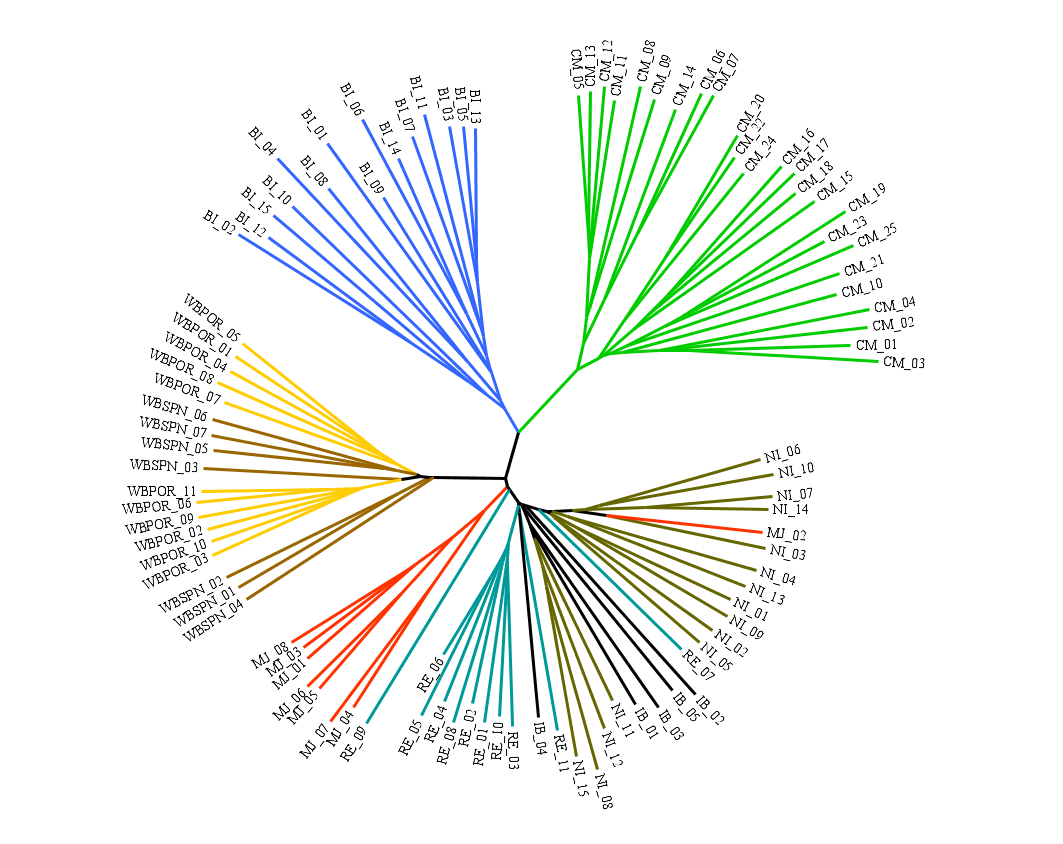

Supplement: Additional file 2 — Neighbor-Joining tree of pig populations constructed from individual pairwise genetic distance. WBPOR, wild boar from Portugal; WBSPN, wild boar from Spain; RE, Retinto Iberian; NI, Negro Iberian, IB, Iberian (unidentified variant); MJ, Manchado de Jabugo; BI, Bisaro; CM, Chato Murciano. [file 1471-2156-14-106-S2.png]

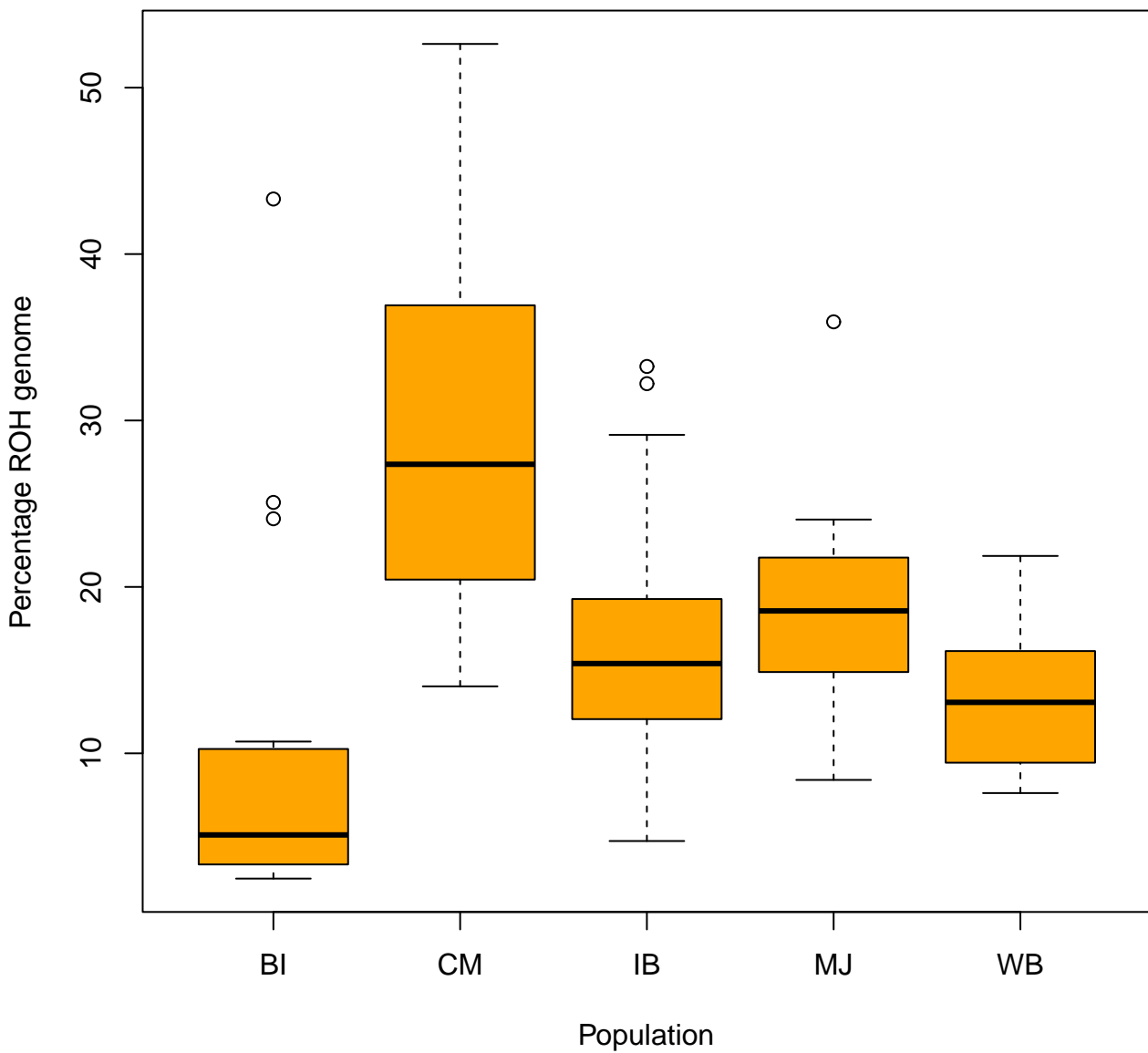

Supplement: Additional file 7 — Estimation of the percentage covered by ROH in each population. WB, wild boar; IB, Iberian; MJ, Manchado de Jabugo; BI, Bisaro; CM, Chato Murciano. [file 1471-2156-14-106-S7.pdf]
