# Supplementary material for: Conservation genomic analysis of domestic and wild pig populations from the Iberian Peninsula
Source: BMC Genet. 2013 Oct 30;14:106. doi: 10.1186/1471-2156-14-106 (PMC3840735; doi:10.1186/1471-2156-14-106)
Supplement: Additional file 4 — STRUCTURE analysis and Evanno method to determine the optimal number of clusters; Membership coefficient of the breeds tested in the four clusters inferred by STRUCTURE software. [file 1471-2156-14-106-S4.pdf]

# This document produced by structureHarvester core version vA.1 March 2012
# http://users.soe.ucsc.edu/~dearl/struct_harvest
# http://taylor0.biology.ucla.edu/structureHarvester
# Written by Dent Earl, dearl (a) soe ucsc edu.
# CITATION:
# Earl, Dent A. and vonHoldt, Bridgett M. (2011)
# STRUCTURE HARVESTER: a website and program for visualizing
# STRUCTURE output and implementing the Evanno method.

## Evanno method

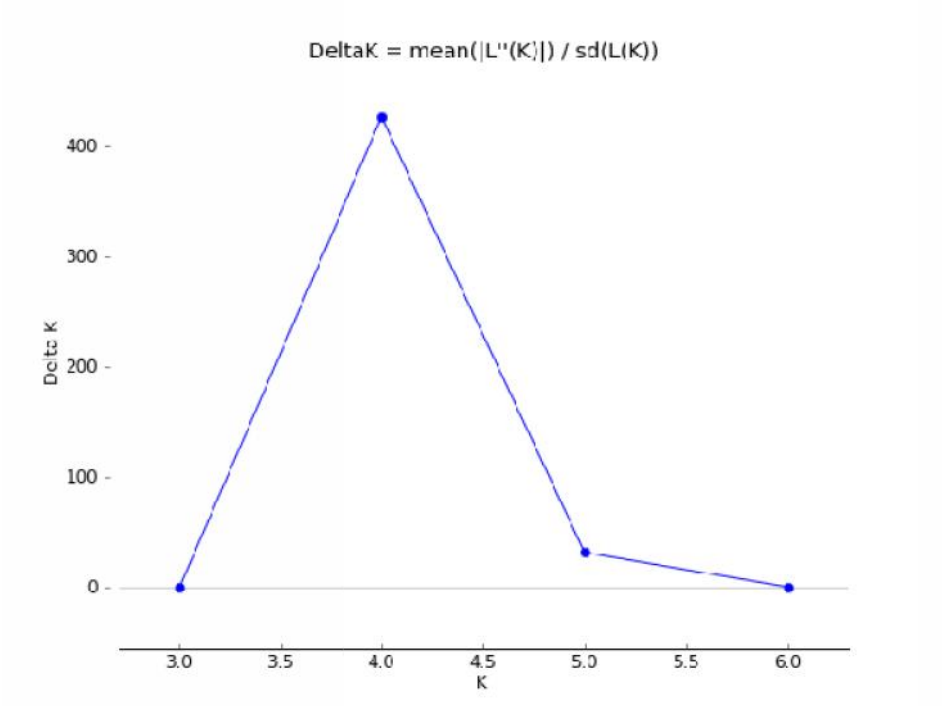


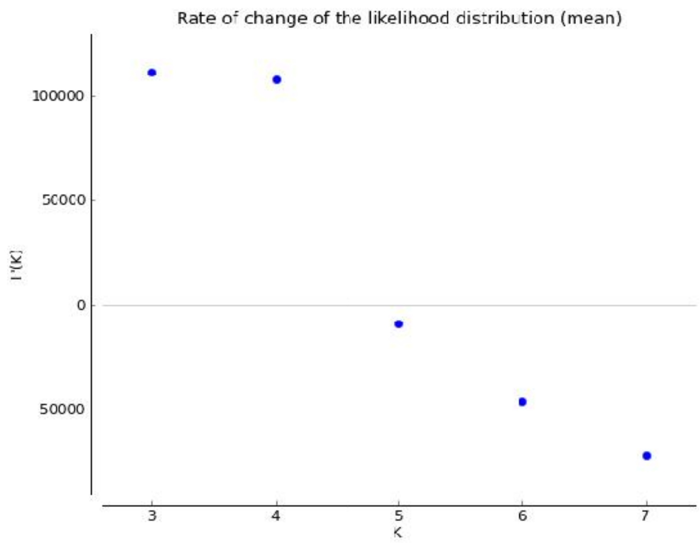

**Evanno table output**

| # K | Reps | mean est. LnP(Data) | stdev est. LnP(Data) |
|---|---|---|---|
| 2 | 2 | -3499745.450000 | 9.545942 |
| 3 | 2 | -3388150.650000 | 22150.756317 |
| 4 | 2 | -3280161.700000 | 274.357431 |
| 5 | 2 | -3289063.750000 | 1175.847866 |
| 6 | 2 | -3335096.900000 | 55106.952778 |
| 7 | 2 | -3406935.300000 | 4731.958580 |

**L(K):**

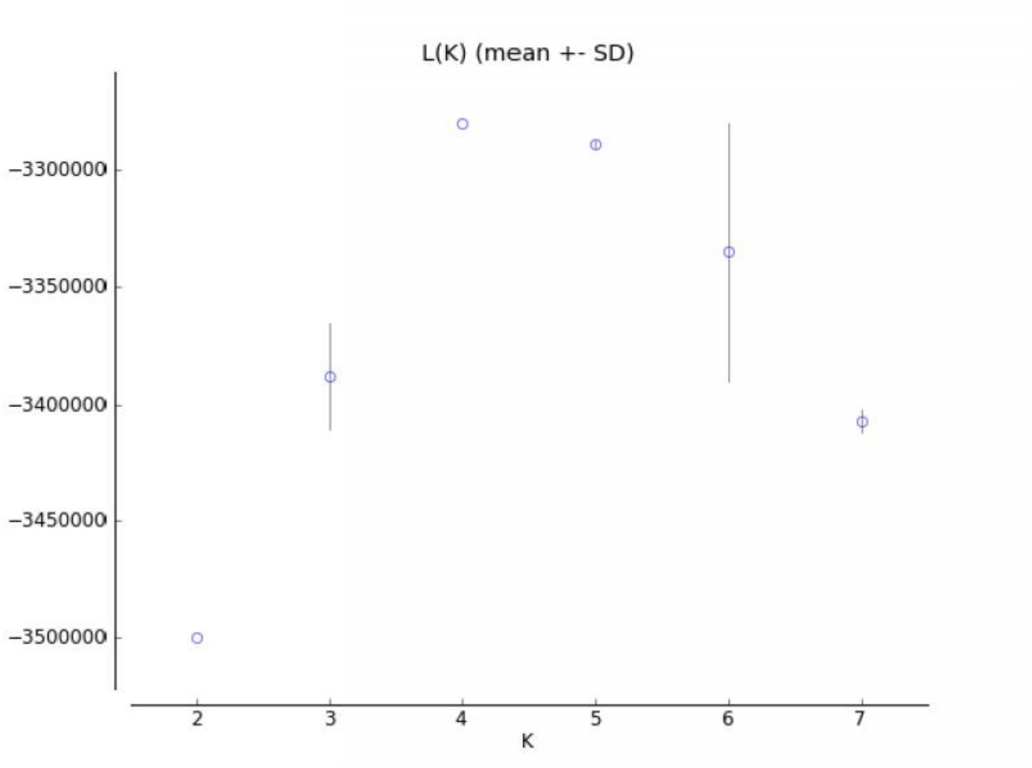


## Raw STRUCTURE output

| # File name | K | Est. Ln prob. of data | Mean value of Ln likelihood | Variance of Ln likelihood |
|---|---|---|---|---|

| | | | | |
|---|---|---|---|---|
| resultsk2_f | 2 | -3499738.7 | -3476196.2 | 47085.0 |
| resultsk2_f | 2 | -3499752.2 | -3476196.9 | 47110.7 |
| resultsk3_f | 3 | -3372487.7 | -3335763.6 | 73448.3 |
| resultsk3_f | 3 | -3403813.6 | -3363514.9 | 80597.4 |
| resultsk4_f | 4 | -3280355.7 | -3224663.5 | 111384.5 |
| resultsk4_f | 4 | -3279967.7 | -3224515.6 | 110904.2 |
| resultsk5_f | 5 | -3289895.2 | -3192130.5 | 195529.5 |
| resultsk5_f | 5 | -3288232.3 | -3191936.2 | 192592.2 |
| resultsk6_f | 6 | -3296130.4 | -3191113.1 | 210034.5 |
| resultsk6_f | 6 | -3374063.4 | -3182128.6 | 383869.6 |
| resultsk7_f | 7 | -3410281.3 | -3180748.0 | 459066.5 |
| resultsk7_f | 7 | -3403589.3 | -3181621.5 | 443935.6 |

**Proportion of membership of each pre-defined population in K = 4:**

| Pop | 1 | 2 | 3 | 4 | Individuals |
|---|---|---|---|---|---|
| WB_Portugal | 0.977 | 0.000 | 0.000 | 0.023 | 11 |
| WB_Spain | 0.878 | 0.000 | 0.000 | 0.122 | 7 |
| Manchado_Jabugo | 0.097 | 0.038 | 0.045 | 0.821 | 8 |
| Negro_Iberico | 0.031 | 0.005 | 0.006 | 0.959 | 15 |
| Retinto | 0.041 | 0.005 | 0.007 | 0.947 | 11 |
| Iberian_Unk | 0.080 | 0.021 | 0.013 | 0.886 | 5 |
| Bisaro | 0.034 | 0.057 | 0.658 | 0.251 | 15 |
| Chato_Murciano | 0.005 | 0.925 | 0.015 | 0.055 | 22 |
